# Intraoperative Molecular Imaging in Thoracic Oncology: Expanding the Observable Disease Space

**DOI:** 10.3390/cancers18142220

**Published:** 2026-07-10

**Authors:** Eliana Marostica, Sunil Singhal

**Affiliations:** Department of Surgery, Hospital of the University of Pennsylvania, Philadelphia, PA 19104, USA; sunil.singhal@pennmedicine.upenn.edu

**Keywords:** intraoperative molecular imaging, fluorescence-guided surgery, thoracic oncology, lung cancer surgery, near-infrared imaging, pulmonary nodules, pafolacianine, fluorescence imaging, image-guided surgery, precision thoracic surgery

## Abstract

Background/Objectives: Intraoperative molecular imaging (IMI) enables real-time visualization of tumor biology during surgery using fluorescent probes and near-infrared imaging systems. As lung cancer screening increases detection of small and nonpalpable pulmonary nodules, conventional localization and margin assessment techniques remain limited, particularly during minimally invasive surgery. This review summarizes the technical foundations, imaging agents, clinical applications, and future directions of IMI in thoracic oncology. Methods: We performed a narrative review to synthesize current evidence regarding the technical foundations, molecular imaging agents, clinical applications, and future directions of intraoperative molecular imaging in thoracic oncology. Given the multidisciplinary scope of the field, a narrative approach was selected to integrate mechanistic, translational, and clinical evidence rather than to answer a single narrowly defined clinical question. Results: IMI generates dynamic intraoperative contrast based on preferential probe accumulation or activation within malignant tissue. Current approaches include non-specific fluorophores such as indocyanine green, activatable probes targeting tumor-associated proteases or acidic microenvironments, and receptor-targeted agents such as pafolacianine. Across prospective studies and multicenter trials, IMI improved localization of nonpalpable lesions, identified occult synchronous malignancies, and enhanced intraoperative margin assessment, frequently altering surgical management. Phase 2 and 3 studies of folate receptor-targeted imaging demonstrated clinically significant findings in a substantial proportion of patients, including lesions not detected by conventional imaging or palpation. However, performance remains dependent on tumor biology, target expression, lesion depth, and optical constraints. Conclusions: IMI represents an emerging transition from anatomy-guided toward biology-informed thoracic surgery by providing real-time molecular information during resection. Current evidence supports its role as a complementary intraoperative technology that augments conventional imaging and surgical techniques, particularly for small, peripheral, and nonpalpable lesions.

## Simple Summary

Lung cancer surgery increasingly involves the removal of very small tumors that can be difficult to see or feel during an operation, especially when minimally invasive techniques are used. Intraoperative molecular imaging is a developing technology that helps surgeons visualize cancer in real time using fluorescent imaging agents and specialized cameras. These agents accumulate within tumors and emit signals that highlight cancerous tissue during surgery. This review summarizes the current state of intraoperative molecular imaging in thoracic oncology, including how the technology works, the imaging agents currently being studied, and its clinical applications for tumor localization, margin assessment, and detection of additional hidden lesions. The findings reviewed suggest that intraoperative molecular imaging may improve surgical precision and help surgeons identify disease that might otherwise be missed using conventional methods alone.

## 1. Introduction

Lung cancer remains the leading cause of cancer-related mortality worldwide, and surgical resection is central to curative-intent treatment for early-stage disease [1,2]. Despite advances in imaging and staging, intraoperative decision-making continues to rely heavily on visual inspection, finger palpation and indirect cues, which incompletely reflect the underlying biology of disease [3,4]. This limitation is particularly evident in lung cancer, where lesions are often small, heterogeneous, and not readily identifiable at the time of resection [5,6]. This reflects a fundamental mismatch between the biological complexity of early lung cancer and the sensory limitations of conventional surgery.

These limitations manifest as persistent intraoperative challenges. Achieving negative margins may be difficult when tumor boundaries are indistinct or subsolid [7,8]. Lesions may be deep within the parenchyma or lack a pleural correlate, and synchronous nodules may remain occult [6,9]. The clinical relevance of these challenges has increased with the widespread adoption of low-dose computed tomography (CT) screening, which has shifted lung cancer toward earlier-stage disease at diagnosis [1]. The National Lung Screening Trial (NLST) demonstrated a 20% relative reduction in lung cancer mortality with low-dose CT screening, with sustained benefit at 12.3 years of follow-up [10,11]. Additionally, the NELSON trial confirmed a 24% mortality reduction in men and 33% reduction in women at 10 years of follow up, establishing screening as standard of care and substantially increasing the detection of small, early-stage nodules [12]. As a result, surgeons are increasingly tasked with managing small pulmonary nodules and ground-glass opacities that are radiographically apparent but difficult to localize intraoperatively [13,14,15]. Conventional approaches based on visual inspection and palpation are less reliable, particularly in the context of minimally invasive surgery, and other pre-operative localization methods have demonstrated increased rates of pneumothorax and hemorrhage [13,14,16,17,18,19].

An emerging technology that has developed over the last two decades to address this challenge is Intraoperative Molecular Imaging (IMI). As compared to conventional imaging modalities that provide static, preoperative anatomical information, IMI generates real-time intraoperative contrast using fluorescent contrast agents and near-infrared imaging systems based on molecular characteristics of tissue [20,21]. This signal arises from the preferential accumulation, binding, or activation of imaging probes within malignant tissue and is interpreted relative to surrounding normal structures.

**Figure 1:**
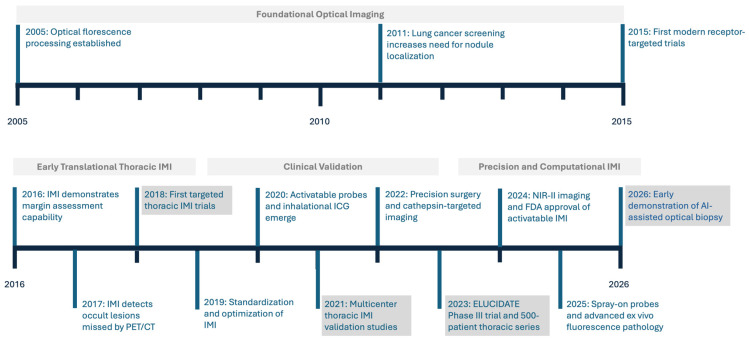
Historical evolution of intraoperative molecular imaging (IMI) in thoracic oncology. Timeline illustrating major conceptual, technical, and clinical milestones in the development of intraoperative molecular imaging for thoracic surgery from 2005 to 2026. Early advances focused on fluorescence signal processing and optical imaging foundations, followed by the emergence of receptor-targeted and activatable imaging probes, thoracic-specific translational studies, and multicenter clinical validation. More recent developments include phase 3 evaluation of pafolacianine-guided surgery, robotic and multispectral imaging integration, ex vivo fluorescence pathology, and early demonstration of artificial intelligence–assisted intraoperative interpretation. Together, these advances reflect the transition of IMI from an experimental optical platform toward a precision surgical technology integrated into thoracic oncologic decision-making.

The clinical utility of IMI depends on both detection and interpretation of fluorescence signal, most commonly quantified using the tumor-to-background ratio (TBR) [2,20]. This framework underpins applications including tumor localization, margin delineation, and detection of occult disease (Figure 1). In the largest single-institution series of 500 pulmonary resections guided by IMI, Kennedy et al. [4] demonstrated that IMI detected positive margins in 6.4% of patients, identified residual disease in 7.4%, and localized nonpalpable lesions in 14.9% of cases. Implementation of IMI requires integration of targeted probes, fluorescence-capable imaging systems, and quantitative analysis within the operative workflow.

In the Phase 3 ELUCIDATE trial, IMI identified the primary lung lesion in 19% of patients where the surgeon could not locate it by white light and finger palpation, and detected occult synchronous malignancies in 8% of participants, underscoring the frequency with which conventional techniques miss clinically significant disease [3]. Frozen section analysis, while widely used, is inherently limited by sampling and processing time [1,22].

In this review, we examine the current state of intraoperative molecular imaging as an approach to augment intraoperative decision-making in lung cancer surgery. We first outline the technical foundations of IMI (Section 2. Technical Foundations), including probe design, imaging systems, and signal quantification. We then review current and investigational imaging agents across non-specific, activatable, and tumor-targeted platforms (Section 3. Molecular Imaging Agents), followed by a discussion of clinical applications and limitations (Section 4. Clinical Applications and Performance). Finally, in Section 5. Discussion and Section 6. Conclusions, we discuss the current limitations of IMI, future directions, and the integration of computational methods to enhance real-time interpretation and expand the role of IMI in precision thoracic oncology.

## 2. Technical Foundations

Intraoperative molecular imaging (IMI) enables real-time visualization of tumor biology during surgery using fluorescent contrast agents and near-infrared (NIR) imaging systems [1,2]. In contrast to static, preoperative conventional imaging modalities, IMI delivers dynamic intraoperative information to the surgeon that reflects molecular characteristics of tissue [1,2].

**Figure 2:**
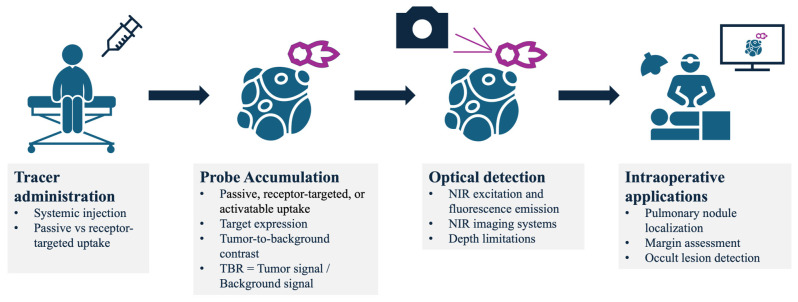
Conceptual framework of intraoperative molecular imaging (IMI) in thoracic oncology. Schematic illustrating the core workflow and biologic principles underlying intraoperative molecular imaging (IMI). Following systemic administration of a fluorescent imaging agent, probes preferentially accumulate within malignant tissue through passive uptake, receptor-targeted binding, or tumor-specific activation mechanisms. Tumor-to-background ratio (TBR) is calculated by dividing tumor fluorescence intensity by adjacent background fluorescence, with higher values generally indicating improved visual contrast. The resulting fluorescence signal is governed by tumor biology, including target expression and tumor-to-background contrast, and is detected intraoperatively using near-infrared (NIR) imaging systems. Optical detection depends on factors such as excitation/emission wavelength, camera sensitivity, and tissue penetration depth. Together, these processes enable real-time intraoperative applications including pulmonary nodule localization, margin assessment, and detection of occult disease during thoracic surgery.

At its core, IMI relies on the preferential accumulation, binding, or activation of a fluorescent probe within malignant tissue. The emitted signal is interpreted relative to surrounding normal tissue using the TBR which governs lesion detectability and clinical utility [23,24]. This approach enables intraoperative identification of lesions, delineation of margins, and detection of otherwise occult disease [3,4,25]. Importantly, IMI performance is a systems-level problem in which TBR emerges from the interaction between probe design, tissue biology, and imaging physics (Figure 2).

### 2.1. Signal-to-Background Ratio and Image Optimization

The clinical utility of IMI depends on achieving sufficient contrast between tumor and surrounding tissue, typically quantified as the signal-to-background ratio (SBR) or tumor-to-background ratio (TBR; used interchangeably) [2,23]. In a robotic simulation study, Azargoshasb et al. [24] demonstrated that task completion time and handling errors increased substantially below an SBR of 1.5, whereas surgical proficiency was achieved only at SBR > 1.55, suggesting that an SBR of approximately 1.5 may be sufficient for reliable visual discrimination under those experimental conditions. However, no universal threshold defines an acceptable TBR or SBR across clinical applications, as measured values depend on the imaging agent, imaging platform, tissue type, fluorescence acquisition parameters, and region-of-interest selection methodology.

SBR is influenced by probe characteristics, dosing, timing of administration, target expression, and imaging system performance [1,23,26]. In a 279-patient retrospective analysis of pafolacianine [OTL38]-guided lung resections, Azari et al. [26] found that patient factors (age, sex, smoking history, infusion-to-surgery interval) did not significantly predict TBR, but distance of the lesion from the organ surface was the dominant determinant (*p* < 0.001). Histologic subtype also matters: adenocarcinoma-spectrum lesions had brighter fluorescence than other NSCLC subtypes (*p* < 0.01) [26]. In the 500-patient institutional series, false-negative fluorescence was primarily seen in mucinous adenocarcinomas (mean TBR 1.8), heavy smokers (>30 pack-years; TBR 1.9), and deep tumors (>2.0 cm from pleural surface; TBR 1.3) [4]. Azari et al. [27] further demonstrated that smoking history and chronic lung disease were correlated with increased background parenchymal fluorescence, likely due to light-absorbing carbon deposition (*p* < 0.05). Optimization therefore requires coordination across pharmacologic and technical domains [1,23].

Multispectral imaging, background subtraction, and targeted excitation can further enhance signal fidelity, particularly under weak fluorescence or high ambient light [28,29,30]. Behrooz et al. [28] demonstrated a multispectral open-air system using synchronized excitation with real-time background subtraction that achieved nanomolar fluorophore sensitivity under full surgical illumination. Waterhouse et al. [29] showed that combining multispectral short-wave infrared imaging with machine learning achieved 97.5% per-pixel classification accuracy for tumor versus non-tumor tissue. These considerations underscore that IMI performance is not solely determined by probe design, but by the interaction between probe, tissue, and imaging system [1,23,31].

### 2.2. Imaging Systems and Equipment

Fluorescence imaging systems incorporate excitation light sources and sensitive detectors capable of capturing low-intensity NIR emission in real time to distinguish signal from surrounding tissue. These platforms are available across open, thoracoscopic, and robotic configurations and are increasingly integrated into standard surgical workflows [32,33].

More than 30 commercially available systems exist, many of which allow simultaneous overlay of fluorescence and white-light imaging, facilitating intuitive interpretation without interrupting operative flow [34]. These systems typically generate real-time color fluorescence overlays superimposed on conventional white-light images, enabling continuous intraoperative visualization without interrupting the surgical workflow. Image resolution, frame rate, and other acquisition parameters vary among commercially available platforms and are largely manufacturer-dependent. For example, Firefly**^®^** for Da Vinci is a robotic-integrated fluorescence imaging system using indocyanine green to primarily assess perfusion and detect biliary structures. Thoracic-specific applications include fluorescence-enabled thoracoscopes and robotic systems, which are particularly relevant in minimally invasive surgery where tactile feedback is limited [32,33,35]. The ability to seamlessly transition between white-light and fluorescence modes is critical, as IMI is most effective when used as an adjunct rather than a replacement for conventional visualization [1,2,36].

### 2.3. Optical Properties and Wavelength Optimization

The detectability of fluorescence signal and resulting TBR are fundamentally determined by the optical properties of tissue and the wavelength of emitted fluorescence. Near-infrared imaging is favored due to reduced tissue autofluorescence, lower photon scattering, and improved depth of penetration relative to visible wavelengths [37,38].

Short-wavelength fluorophores (visible to NIR-I) are limited by shallow penetration and higher background signal, whereas longer-wavelength agents demonstrate improved tissue penetration and signal-to-background characteristics [39]. Clinically, this translates to meaningful differences in detection depth—on the order of millimeters for short-wavelength agents versus up to 1–2 cm for longer-wavelength tracers. For example, in a thoracic comparative study by Kennedy et al. [39], the limit of signal detection was 1.8 cm from the pleural surface for pafolacianine (λem = 793 nm) compared to only 0.3 cm for folate-fluorescein (λem = 520 nm), with pafolacianine having a significantly higher mean signal-to-background ratio (2.71 vs. 1.73, *p* < 0.0001). In the Phase 3 ELUCIDATE trial, the depth of primary lung lesions detected by pafolacianine ranged from 0 to 38 mm from the lung surface [3,40].

Emerging imaging in the NIR-II spectrum further reduces scattering and background interference, offering improved spatial resolution and deeper tissue interrogation [41,42]. In a clinical comparison of ICG fluorescence in NIR-I versus NIR-II windows, Mi et al. [43] demonstrated that NIR-II achieved a significantly higher tumor-to-normal-tissue ratio than NIR-I (3.9 ± 1.3 vs. 2.4 ± 0.6, *p* < 0.001), with superior signal-to-background ratio when lung tissue thickness exceeded 2 mm. Starosolski et al. [42] similarly showed that ICG contrast-to-noise ratios in the NIR-II window were approximately 2-fold higher than in the NIR-I window. However, fluorescence imaging remains inherently depth-limited. In thoracic applications, reliable detection is typically confined to lesions within approximately 5–10 mm of the pleural surface, although lung deflation during surgery may effectively reduce this distance, as the collapsed lung undergoes volumetric changes that bring tumors closer to the surface than measured on preoperative CT [4,44]. Despite these promising advances, the absence of FDA-approved clinical imaging systems capable of NIR-II fluorescence imaging remains a significant barrier to routine implementation in thoracic surgery. Current clinically translated thoracic IMI agents almost exclusively operate in the NIR-I window, whereas NIR-II imaging remains an emerging platform that will likely require development of new fluorophores and dedicated imaging hardware before routine clinical implementation.

### 2.4. Real-Time and Ex Vivo Imaging

A defining feature of IMI is its ability to provide both intraoperative and ex vivo information [1,2]. Following systemic administration of a fluorescent tracer—typically hours to days prior to surgery—tumors can be visualized in real time during resection, enabling guidance of lesion localization and margin assessment [3,4]. In the Phase 3 ELUCIDATE trial, real-time IMI with pafolacianine localized the primary lung lesion in 19% of patients where the surgeon could not locate it with white light and palpation, and identified close margins (≤10 mm) in 38% of evaluated participants [3]. In the 500-patient institutional series by Kennedy et al. [4], IMI detected positive margins in 6.4% of patients, identified residual disease in 7.4%, and localized nonpalpable lesions in 14.9% of cases.

Resected specimens can subsequently undergo ex vivo fluorescence imaging for rapid margin assessment and targeted frozen section guidance [2,6]. In controlled closed-field environments, ex vivo imaging yields higher TBRs than in vivo imaging and can correlate fluorescence intensity with margin distance, effectively generating a fluorescent map of the specimen [6]. Kennedy et al. [5] demonstrated near-perfect correlation between IMI-derived and pathology-determined margin distances in resected lung specimens, enabling near real-time assessment of margin adequacy. Additional approaches such as ex vivo fluorescence confocal microscopy have further demonstrated accurate identification of tumor-involved staple lines and margins with results concordant with paraffin histopathology [7,8].

Emerging approaches extend ex vivo imaging beyond margin assessment toward rapid intraoperative optical biopsy. Li et al. [9] showed that topical application of an EGFR-targeted NIR-II fluorescent probe to freshly resected lung tissue achieved 85.7% sensitivity and 100% specificity for identifying tumor areas following a 20 min incubation, with a mean TBR of 2.5 ± 1.3. Deutsch-Williams et al. [22] demonstrated that FAP-targeted spray-on probes applied topically to resected specimens increased tumor margin contrast by 5- to 10-fold compared to systemic agents and detected even microscopic cancer deposits within minutes. This approach remains preclinical/early clinical and requires further validation in thoracic surgery. Together, real-time and ex vivo imaging provide complementary information: the former guides surgical decision-making, while the latter harnesses increased TBR to refine pathologic assessment and margin evaluation [1,2,6].

### 2.5. AI-Augmented Intraoperative Molecular Imaging

Computational approaches may further enhance IMI through real-time fluorescence interpretation and optical biopsy. Azari et al. [45] developed and prospectively validated a machine learning algorithm that combines image segmentation–based fluorescence quantification with a clinical nomogram to perform real-time “optical biopsy” during IMI-guided lung cancer surgery. In a cohort of 322 patients, the algorithm achieved an AUC of 0.865–0.893 for malignant nodule assessment, and prospective validation in 61 consecutive patients demonstrated 93.8% sensitivity, 100% specificity, and 100% positive predictive value for identifying malignant lung nodules [45]. Critically, the algorithm determined malignant potential in a mean of 1.8 min compared with 34 min for frozen section analysis, suggesting that AI-augmented IMI could substantially accelerate intraoperative decision-making while reducing reliance on traditional pathology workflows [45].

## 3. Molecular Imaging Agents

Molecular imaging in surgery can be broadly categorized based on the mechanism by which contrast agents achieve tumor specificity. These include (i) non-specific tumor-targeted agents, (ii) activatable probes, and (iii) receptor-targeted agents (Table 1). Each class reflects a distinct biological strategy—passive accumulation, microenvironment-triggered activation, or ligand-receptor binding—and carries unique advantages and limitations with respect to sensitivity, specificity, and clinical translation [1,21]. The imaging agents reviewed in this section have generally demonstrated acceptable safety profiles in clinical studies, although agent-specific adverse events, dosing considerations, regulatory approval status, and stage of clinical development differ substantially across platforms. These factors should be considered alongside imaging performance when evaluating their potential for clinical translation.

### 3.1. Non-Specific Tumor-Targeted Agents

Indocyanine green (ICG) remains the most widely utilized fluorophore in clinical practice due to its favorable safety profile, FDA approval, and compatibility with near-infrared (NIR) imaging systems [1,46]. While originally developed for angiography and perfusion assessment, its role in oncologic imaging has expanded through exploitation of the enhanced permeability and retention (EPR) effect, whereby ICG passively accumulates in tumors due to leaky vasculature and impaired lymphatic drainage [46]. Mechanistic studies have demonstrated that ICG tumor uptake involves both EPR-mediated extravasation and active cellular endocytosis via clathrin-mediated pathways, with intracellular retention in lysosomes contributing to prolonged tumor signal [47].

The “TumorGlow” technique represents a paradigm shift in the use of ICG for intraoperative tumor visualization. By administering high-dose ICG (typically 2–5 mg/kg) 24 h prior to surgery, preferential tumor accumulation is achieved, enabling intraoperative detection of malignant lesions [48,49]. Dose-optimization studies demonstrated histology-dependent performance, with lower ICG doses favoring non-primary lung cancers and higher doses improving visualization of primary NSCLC at the expense of increased background fluorescence [48]. This approach has demonstrated particular utility in thoracic oncology, including pulmonary metastases and primary lung cancers. Across the 500-patient institutional series by Kennedy et al. [4], TumorGlow was most effective for metastatic disease and mesothelioma (mean TBR 3.1). Jeon et al. [50] confirmed in a 51-patient clinical study that ICG at 2 mg/kg administered 12 h before surgery successfully detected 37 of 39 lung cancers with a consolidation-to-tumor ratio > 50% (detection rate 95%, mean TNR 3.3 ± 1.2), but failed in lesions with a C/T ratio ≤ 50%.

**Table 1:** Summary of representative intraoperative molecular imaging agents evaluated in thoracic oncology.

Agent	Class	Clinical Evidence	Patients (*n*)	Localization	Margins	Occult Lesions	CSE	TBR/SBR	Wavelength	Major Limitations
Pafolacianine (OTL38) [3,4,25]	FRα	Ph2/Ph3; 500-pt	92/100/500	19%	38% close	8–10%	26–53%	2.84	793 nm (NIR-I)	Lower signal in mucinous tumors, smokers, deep lesions; variable FRα in SCC
EC17 [4,39]	FRα	Comparative	71	Limited (3 mm)	NR	NR	NR	SBR 1.73	520 nm (visible)	Autofluorescence; shallow penetration
ICG (TumorGlow) [48]	EPR	Dose optimization	45	+500 subset	NR	NR	NR	2.7–3.1	800–830 nm (NIR-I)	Non-specific uptake; inflammation/fibrosis
ICG (Inhaled) [44,51]	Negative contrast	RCT/Series	56/43	87%	NR	NR	NR	BTR 7.1	800–830 nm (NIR-I)	≤1 cm depth; investigational
Abenacianine (VGT-309) [52,53,54]	Cathepsin	Ph2	40/89/27	38%	9%	2%	43–45%	NR	800–820 nm (NIR-I)	Early phase; transient transaminitis
Pegsitacianine (ONM-100) [55,56]	pH	Ph2	20	Poor	NR	NR	NR	2.4–2.7 (pilot)	820 nm (NIR-I)	Sensitivity 32%; specificity 33%; infusion reactions
SGM-101 [4,57]	CEACAM5	Ph1	Small	NR	NR	NR	NR	3.11	700 nm (NIR-I; BM-104)	Limited lung data; delayed imaging
Cetuximab-IRDye800CW [58,59,60]	EGFR	Ph2 ongoing	3	NR	Ex vivo feasible	NR	NR	1.4× contrast	800 nm (NIR-I)	Limited lung data; heterogeneous EGFR
Panitumumab-IRDye800CW [61]	EGFR	Pilot	NR	NR	Ex vivo feasible	NR	NR	Lower than HNSCC	800 nm (NIR-I)	No dedicated lung trial

Abbreviations: CSE = clinically significant event; TBR = tumor-to-background ratio; SBR = signal-to-background ratio; BTR = background-to-tumor ratio; NR = not reported; HNSCC = head and neck squamous cell carcinoma; Ph1 = Phase 1; Ph2 = Phase 2; Ph3 = Phase 3. Note: Outcomes are reported only where evaluated in the original studies. Direct comparison across agents should be interpreted cautiously because study populations, imaging systems, outcome definitions, and trial designs differ substantially.

Beyond thoracic applications, TumorGlow has been applied to sarcoma resections. Nicoli et al. [62] reported the first use of NIR fluorescence guidance in open sarcoma surgery, demonstrating fluorescence in the majority of treatment-naive high-grade sarcomas and guiding further tissue resection to improve margins in 3 of 11 cases. Brookes et al. [63] subsequently showed in a larger series that patients receiving ICG-guided surgery had a significantly lower unexpected positive margin rate compared to conventional surgery (5.1% vs. 25.0%, *p* = 0.01). Schermerhorn et al. [64] demonstrated the utility of ICG for pulmonary metastasectomy in pediatric and young adult sarcoma patients, with an overall sensitivity of 81% and identification of 17% of metastases not visible by white light or identifiable with palpation. Retrospective analyses have also suggested a potential association between IMI-guided pulmonary metastasectomy and improved progression-free survival, although these findings remain hypothesis-generating. In a hypothesis-generating study of pulmonary sarcoma metastasectomy, IMI-guided resection was associated with longer median progression-free survival (36.0 vs. 28.6 months; *p* < 0.05), although hazard ratios and confidence intervals were not reported. IMI-guided pulmonary sarcoma metastasectomy had recurrence in the lung with a median time of 18 months compared with non-IMI group at 13 months (*p* < 0.05) [65]. Though limited by risk of selection bias and retrospective analysis, these findings support further prospective evaluation, including randomized studies.

More recently, alternative delivery strategies such as inhalational ICG administration have been explored, particularly for lung-specific applications [44,51]. Quan et al. [44] demonstrated in JAMA Surgery that ICG inhalation achieved tumor margin detection efficiency 2-fold higher than intravenous injection at a 20-fold lower dose (0.25 mg/kg inhaled vs. 5.0 mg/kg IV, *p* < 0.001), with tumors as small as 0.2 cm detectable within 10 min to 24 h after inhalation. In practice, inhalational ICG produces a “negative contrast” image—healthy lung tissue fluoresces while tumor tissue appears dark due to airflow obstruction and alveolar destruction—which is mechanistically distinct from the EPR-based “positive contrast” of intravenous ICG [44]. Wang et al. [51] confirmed clinical feasibility in 43 patients, detecting 44 of 50 pulmonary nodules with a median background-to-tumor ratio of 7.10 and median detection time of 100 s, with no adverse events. Huang et al. [66] reported a clinical case demonstrating successful thoracoscopic localization of a ground-glass opacity using ICG inhalation performed 85 min before surgery, with the fluorescence filling defect clearly delineating the tumor margin. Although still early in development, inhalational techniques may improve tumor-to-background ratios and enable real-time intraoperative imaging. However, clinical experience remains limited, and optimal dosing, timing, and delivery parameters are not yet well established.

Despite its accessibility, ICG-based imaging is fundamentally limited by its lack of tumor specificity [1]. False positives can arise from inflammation, fibrosis, or other processes associated with increased vascular permeability, which can prompt unnecessary tissue sampling, additional wedge resections, or prolonged intraoperative evaluation to exclude malignancy. False negatives have also arisen in mucinous adenocarcinomas (mean TBR 1.8), heavy smokers (>30 pack-years; TBR 1.9), and tumors greater than 2.0 cm from the pleural surface (TBR 1.3) [4]. As such, while non-specific agents offer ease of use and broad applicability, they are inherently constrained in their ability to discriminate malignant from benign tissue and should be interpreted in conjunction with preoperative imaging, anatomic landmarks, and clinical judgment rather than as independent evidence of malignancy.

### 3.2. Activatable Probes

Activatable probes represent a more sophisticated approach to tumor imaging, leveraging the unique biochemical features of the tumor microenvironment to generate fluorescence only in the presence of disease [1]. Unlike always-on fluorophores such as ICG, these agents remain optically silent until activated by specific stimuli, thereby enhancing signal-to-background contrast.

Pegulicianine (Lumisight) is among the most clinically advanced activatable probes and serves as an important proof-of-concept for fluorescence-guided surgery [21]. This protease-activated agent remains optically quenched until cleavage by tumor-associated enzymes, enabling real-time identification of residual tumor at surgical margins. Early clinical studies demonstrated favorable safety and tumor-specific fluorescence [67], while subsequent feasibility and multicenter trials showed improved detection of residual disease and reduced re-excision rates following lumpectomy [68,69,70]. In the pivotal 406-patient multicenter trial, pegulicianine-guided surgery prevented second operations in 15% of patients with positive margins [69], ultimately leading to FDA approval in 2024 for intraoperative detection of cancerous tissue during breast-conserving surgery [21,71].

pH-activated probes constitute another important subclass, exploiting the acidic microenvironment characteristic of many solid tumors. Pegsitacianine (ONM-100, OncoNano) is designed to undergo nanoparticle disassembly at low pH, resulting in fluorescence activation specifically within tumor tissue [72]. Voskuil et al. [72] demonstrated in the first-in-human study that ONM-100 was well tolerated in 30 subjects across four solid tumor types, assisted in detection of tumor-positive resection margins in 9/9 subjects, and facilitated identification of four additional occult lesions that otherwise would have been missed. Steinkamp et al. [73] subsequently validated a standardized specimen-driven fluorescence framework using ONM-100 in head and neck squamous cell carcinoma, detecting all six tumor-positive surgical margins with a median TBR of 3.36. Kennedy et al. [55] showed that pegsitacianine could label both adenocarcinoma and squamous cell carcinoma of the lung (TBRs = 2.7 and 2.4, respectively). However, in the subsequent Phase 2 multicenter lung cancer trial, pegsitacianine achieved only 31.6% sensitivity and 33.3% specificity, with only 6 of 19 malignant lesions fluorescing, suggesting that lung tumors may not be as acidic as other solid tumors [56]. This negative trial illustrates an important biological principle. Unlike head and neck cancers, lung tumors may not consistently develop sufficiently acidic extracellular microenvironments to activate pH-sensitive probes, emphasizing that successful IMI depends on disease-specific biology rather than simply probe design. This mechanism enables broad tumor applicability in principle, as extracellular acidity is a common feature across malignancies independent of specific receptor expression, but the lung cancer data highlight the importance of tumor-type-specific validation.

Protease-targeted probes, particularly those activated by cathepsins, have also gained significant attention. Cathepsins are lysosomal proteases that are frequently upregulated and secreted in tumor-associated macrophages and cancer cells. Abenacianine [VGT-309] is a quenched activity-based probe that fluoresces upon covalent binding to active cathepsins. Kennedy et al. [74] reported the first-in-human study demonstrating safety across all doses and successful localization of visually occult, non-palpable lung tumors (TBRs = 2.83 and 7.18). In the single-center Phase 2 trial of 40 patients, Bou-Samra et al. [52] found that 42.5% had at least one clinically significant event, including localization of lesions not found by standard methods in 16 patients. The Phase 2 multicenter trial (89 participants) presented at ASCO 2025 confirmed these findings, with 45% of participants having at least one clinically significant event, including lesion localization in 38%, margin assessment in 9%, and detection of synchronous cancers in 2% [53]. Wright et al. [54] established the optimal dosing at 0.32 mg/kg administered 12–36 h before surgery in a Phase 2 dose-ranging trial. Robotic IMI with abenacianine was also demonstrated to achieve 88.9% sensitivity, equivalent to VATS-based IMI, with a mean TBR of 9.20 during robotic imaging [75].

Overall, activatable probes offer a compelling balance between specificity and versatility. However, their performance is dependent on the presence and activity of the target biological process, which may vary across tumor types and patients. Furthermore, regulatory and manufacturing complexities have thus far limited widespread clinical adoption.

### 3.3. Receptor-Targeted Agents

Receptor-targeted imaging agents achieve specificity through direct binding to cell surface or extracellular targets overexpressed in tumors. These agents can be broadly categorized into peptides and proteins, antibodies, and small molecules, each with distinct pharmacokinetic and imaging characteristics.

Antibody-based imaging agents offer high specificity due to their strong affinity for tumor-associated antigens. One of the most well-studied targets is CEACAM5 (carcinoembryonic antigen-related cell adhesion molecule 5), which is overexpressed in a variety of adenocarcinomas, including colorectal and pancreatic cancers [76]. SGM-101, a fluorescently labeled anti-CEACAM5 antibody, has demonstrated promising results in clinical trials. In the first-in-human dose-escalation pilot study by Boogerd et al. [76], SGM-101 was safe and achieved 98% sensitivity and 84% diagnostic accuracy in the expansion cohort, with fluorescence imaging detecting 43% of lesions not clinically suspected that subsequently altered treatment strategy in 35% of patients. Azari et al. [57] demonstrated in a Phase 1 proof-of-principle trial that SGM-101 localized to CEACAM5-positive lung tumors, with patients that exhibited 2+ and 3+ tissue CEACAM5 expression achieving a mean TBR of 3.11. Notably, lack of fluorescence was associated with benign lesions and absence of CEACAM5 staining [57].

Similarly, epidermal growth factor receptor (EGFR)–targeted agents have been developed for use in tumors such as head and neck squamous cell carcinoma and glioblastoma. In a 2015 dose-escalation study in head and neck cancer, Rosenthal et al. demonstrated that cetuximab-IRDye800CW was safe, achieving a mean TBR of 5.2 at the highest dose level, with fluorescence intensity positively correlating with EGFR expression [77]. Cetuximab-IRDye800CW also yielded 97% sensitivity and 93% specificity for ex vivo detection of lymph node metastases, with deeper sectioning of fluorescently false-positive nodes revealing occult metastases in 8.1% of cases [78]. Panitumumab-IRDye800CW, a fully humanized anti-EGFR antibody conjugate with an improved safety profile, has shown similar promise; Gao et al. [79] demonstrated comparable pharmacodynamic properties and minimal toxicities for both agents in parallel Phase 1 trials, with no grade 2 or higher toxicities attributable to either conjugate. In oropharyngeal cancer specifically, Stone et al. [80] reported interim Phase 2 results showing that panitumumab-IRDye800CW delineated tumors from normal tissue during transoral robotic surgery with a mean TBR of 10.7, including in tumors with low EGFR expression, and identified a tumor fragment initially overlooked on standard white-light views. These agents leverage the overexpression of EGFR to enhance tumor visualization, although variability in receptor expression and potential background uptake in normal tissues remain challenges.

While antibodies provide excellent specificity, their relatively large size results in prolonged circulation times and delayed imaging windows, often requiring administration days prior to surgery. Efforts to develop smaller antibody fragments and peptide-based agents aim to address these limitations by improving tissue penetration and enabling same-day imaging. In contrast, small molecule agents offer rapid pharmacokinetics, favorable tissue penetration, and the potential for same-day administration, making them particularly attractive for clinical use. Among these, folate receptor-targeted agents represent one of the most successful translational examples.

Pafolacianine [OTL38] is an FDA-approved NIR fluorophore that targets folate receptor-α (FRα), which is overexpressed in ovarian cancer and other epithelial malignancies [40,80,81]. By conjugating a folate analog to a NIR dye, pafolacianine enables highly specific binding and internalization into tumor cells via receptor-mediated endocytosis. Predina et al. [82,83] confirmed FRα expression in 86% of pulmonary adenocarcinomas and demonstrated 100% tumor identification in a Phase 1 trial of 20 patients, with a mean TBR of 2.9 and detection of four subcentimeter nodules not identified on preoperative imaging. In the Phase 2 multicenter trial (92 evaluable patients), 26% of patients had improvement in outcomes, including detection of 9 occult cancers (10%), localization of 11 lesions unfindable by the surgeon (12%), and identification of 8 positive margins (9%) [25]. The pivotal Phase 3 ELUCIDATE trial (100 evaluable participants) demonstrated clinically significant events in 53% of participants (*p* < 0.0001), including close margins in 38%, primary nodule localization in 19%, and detection of 10 occult synchronous malignancies in 8% [3]. A subsequent analysis of the ELUCIDATE data by Rice et al. [81] showed that IMI identified 29 occult lesions in 23% of participants, of which 34% were malignant, including 7 synchronous stage I lung cancers.

Of note, earlier-generation agents such as EC17 (combines folate with fluorescein) operate in the visible spectrum and are therefore limited by increased tissue autofluorescence and reduced penetration depth [39]. In contrast, NIR agents like pafolacianine offer superior imaging characteristics, including deeper tissue penetration and improved signal-to-background ratios. Kennedy et al. [39] directly compared these agents in 282 patients and found pafolacianine achieved a significantly higher mean SBR (2.71 vs. 1.73, *p* < 0.0001) and 6-fold greater depth of detection (1.8 cm vs. 0.3 cm).

Importantly, folate receptor-targeted imaging is influenced by differential expression of FRα and FRβ. While FRα is primarily expressed on tumor cells, FRβ is found on activated macrophages, which may contribute to signal in inflammatory or immune-rich environments. Azari et al. [84] prospectively validated that increased FRα expression on preoperative core biopsy was significantly associated with intraoperative fluorescence (*p* = 0.01), with adenocarcinomas achieving a mean TBR of 3.11 compared to 1.89 for squamous cell carcinomas (*p* < 0.01). Sarkaria et al. [85] confirmed that 92% of lesions tested across the combined Phase 2/3 trials were positive for FRα or FRβ expression, and that pafolacianine targets a broad histological cross-section including adenocarcinoma, squamous cell carcinoma, adenoid cystic carcinoma, and metastatic lesions.

Taken together, these three classes of molecular imaging agents reflect an evolution from passive accumulation to biologically informed targeting strategies. Non-specific agents such as ICG provide accessibility and broad applicability but lack precision. Activatable probes introduce conditional specificity based on tumor biology, while receptor-targeted agents offer high specificity at the cost of increased complexity and potential variability in target expression. Future advances will likely involve hybrid approaches that integrate these mechanisms, alongside improvements in fluorophore chemistry and imaging system sensitivity, to further enhance intraoperative decision-making.

Although much of the prospective clinical evidence in thoracic IMI has originated from a limited number of high-volume centers and collaborating multicenter investigations, important complementary contributions have emerged from independent research groups. Alternative imaging strategies—including inhaled ICG, EGFR-targeted probes, NIR-II fluorescence imaging, and fluorescence-assisted pathology—have expanded the biologic and technological landscape of thoracic IMI while highlighting the diversity of approaches under investigation. Studies evaluating inhaled ICG have demonstrated promising localization of superficial pulmonary nodules, whereas investigations of EGFR-targeted imaging and NIR-II fluorescence have established technical feasibility but remain at earlier stages of clinical validation. Collectively, these complementary efforts reinforce that successful clinical translation depends not only on fluorescence technology itself, but also on appropriate target selection, tumor biology, and continued multicenter validation across diverse patient populations and imaging platforms.

## 4. Clinical Applications and Performance

As introduced above, intraoperative molecular imaging (IMI) is best understood not as a standalone imaging modality, but as an intraoperative adjunct designed to overcome the fundamental limitations of vision, palpation, and preoperative imaging in thoracic surgery. Its clinical utility therefore lies in how effectively it augments real-time surgical decision-making—specifically in lesion localization, margin assessment, and the identification of otherwise occult disease. Clinical studies discussed in this review include Phase 1 (safety and feasibility), Phase 2 (preliminary efficacy and optimization), and Phase 3 (large-scale comparative validation) clinical trials.

The most immediate application of IMI is in the localization of nonpalpable pulmonary nodules, an increasingly common challenge in minimally invasive thoracic surgery. Small, soft, or ground-glass lesions are frequently identifiable on preoperative imaging yet remain difficult to detect intraoperatively due to the loss of tactile feedback [14,86]. Multiple prospective studies have demonstrated that IMI improves localization of nonpalpable pulmonary nodules, particularly subcentimeter and ground-glass lesions that are difficult to identify during minimally invasive surgery. Across prospective clinical studies, IMI consistently facilitated localization of lesions that could not be identified by conventional visualization or palpation [3,4,5,25,87,88], thereby reducing the gap between preoperative detection and intraoperative identification. Representative trial outcomes are summarized in Table 1.

Despite these encouraging results, IMI should be considered within the broader landscape of existing localization strategies rather than as a replacement for established techniques (Table 2). Conventional approaches—including CT-guided hook wire or microcoil placement [89,90], radioguided techniques [91,92,93,94], electromagnetic navigation bronchoscopy [95,96], transthoracic dye localization [97], and intraoperative ultrasound [17]—remain widely used and have demonstrated high localization success in appropriately selected patients [19,98,99,100,101]. However, these techniques generally require additional preoperative procedures, specialized equipment, or substantial operator expertise, and most are designed solely to facilitate lesion localization without providing information regarding tumor biology, margin status, or occult synchronous disease. In contrast, IMI offers real-time molecular contrast that can simultaneously assist with lesion localization, intraoperative margin assessment, and detection of otherwise occult malignant lesions. As such, IMI is best viewed as a complementary adjunct to existing localization strategies, with the potential to enhance intraoperative decision-making while integrating with established surgical workflows.

In parallel, IMI has also demonstrated value for intraoperative margin assessment, with prospective studies showing improved identification of close or positive margins that altered surgical management, particularly during sublobar resection [3,4,5,25,102]. The broader clinical importance of these findings is underscored by the established association between positive margins and inferior oncologic outcomes following NSCLC resection. For example, positive margins following NSCLC resection occur in approximately 5–15% of patients undergoing curative-intent procedures and are associated with inferior long-term outcomes through local and distant recurrence [103]. By enabling real-time intraoperative margin assessment, IMI may improve the ability to achieve complete oncologic resection while potentially reducing the need for reoperation.

Beyond localization and margin assessment, IMI also reveals a layer of disease that lies beyond the resolution of current preoperative imaging. Across multiple prospective studies, IMI consistently identified occult synchronous pulmonary lesions not detected by preoperative imaging or conventional intraoperative assessment. These additional lesions frequently resulted in disease upstaging, modification of the planned operation, or additional wedge resections. Although the frequency of occult lesion detection varied according to imaging agent and study population, this observation has now been reported across receptor-targeted and activatable probes, suggesting that detection of otherwise occult disease represents one of the most clinically impactful applications of IMI.

This capability is well illustrated by a prospective 50-patient study directly comparing IMI with FDG-PET/CT, in which IMI identified nine additional malignant lesions not detected preoperatively, all of which were significantly smaller than lesions identified by conventional imaging (0.5 vs. 2.4 cm, *p* < 0.01) [104]. In addition, sensitivity was superior compared with PET (95.6% vs. 73.5%, *p* = 0.001), resulting in clinical upstaging in six patients (12%) and altered surgical management in 15 patients (30%) [104].

Collectively, prospective clinical studies support IMI as a valuable adjunct for intraoperative decision-making by improving localization of nonpalpable nodules, facilitating real-time margin assessment, and identifying otherwise occult malignant lesions. Although the magnitude of benefit varies according to imaging agent, target biology, and clinical setting, these findings demonstrate the broad clinical potential of IMI across multiple thoracic applications. Future multicenter studies using standardized imaging protocols, fluorescence quantification methods, and outcome definitions will be important to facilitate more rigorous comparison across imaging agents and to define the patient populations most likely to benefit from IMI.

## 5. Discussion

Taken together, the technical foundations and clinical applications reviewed above demonstrate that IMI is best understood as a systems-level technology in which clinical utility emerges from the interaction between tumor biology, probe design, and optical constraints. Its performance is therefore not uniform across disease states, but instead reflects the degree to which these variables align to produce sufficient tumor-to-background contrast for reliable intraoperative interpretation. As a narrative review, this manuscript is intended to provide a broad synthesis of the field rather than a systematic appraisal of all available studies. Consequently, selection bias is an inherent limitation, although this approach permits integration of technical, biologic, and clinical concepts that would be difficult to capture within a traditional systematic review framework.

The results reviewed here suggest that IMI does more than improve visualization—it exposes intrinsic limitations in current surgical and imaging paradigms. The consistent detection of occult synchronous malignancies and unanticipated positive or close margins across multiple studies indicates that a subset of clinically relevant disease remains systematically undetected by standard approaches. In this context, IMI functions not only as an adjunct, but as a modality that expands the observable disease space within the operating room.

The extent to which IMI can reveal this otherwise occult disease depends largely on the biological specificity of the imaging strategy employed. The relationship between tumor biology and intraoperative signal generation is most clearly illustrated by the clinical experience with folate receptor–targeted imaging. The strong performance of pafolacianine in lung cancer is not incidental, but rather reflects the high prevalence of folate receptor-α expression in pulmonary adenocarcinomas, with reported positivity rates exceeding 90% in large prospective cohorts. This biologic enrichment directly translates to higher tumor-to-background ratios and more consistent intraoperative signal, explaining why the most robust clinical data—including phase 2/I3 trials and large institutional series—have been observed in this histologic subtype. At the same time, emerging receptor-targeted approaches such as CEACAM5-directed imaging extend this paradigm but also emphasize a central limitation: tumor-specific expression is heterogeneous across lung cancer subtypes and metastatic lesions, and no single target is likely to provide universal applicability.

Although much of the prospective thoracic IMI literature has originated from the University of Pennsylvania and collaborating multicenter trials, independent validation remains comparatively limited. Independent groups have contributed important work in inhaled ICG, NIR-II imaging, fluorescence pathology, and EGFR-targeted imaging, although these approaches generally remain at earlier stages of clinical validation. Broader multicenter validation across institutions, imaging platforms, imaging systems, and patient populations will ultimately be necessary to establish the generalizability of current findings and define the role of IMI across diverse practice settings.

An important observation from the broader thoracic literature is that not all fluorescence-guided approaches have translated equally well to clinical practice. While receptor-targeted agents such as pafolacianine and CEACAM5-directed probes have demonstrated consistent clinical benefit, alternative strategies including passive intravenous indocyanine green, inhalational fluorescence, EGFR-targeted antibodies, and pH-activatable probes have produced more variable results. In some cases, limited penetration depth, heterogeneous target expression, or insufficient biologic specificity have restricted clinical performance despite encouraging preclinical data. Conversely, inhaled ICG has shown promising localization of superficial pulmonary nodules but remains constrained by limited depth and lack of tumor specificity. Collectively, these experiences illustrate that successful translation depends not only on fluorescence technology itself but on alignment between tumor biology, probe pharmacology, and optical physics (Table 1).

The limitations of IMI similarly reflect the combined influence of tumor biology and optical physics. False-negative fluorescence is most commonly observed in tumors with low or variable target expression, including mucinous adenocarcinomas, as well as in patients with heavy smoking histories, where increased background parenchymal signal reduces tumor-to-background contrast. These biologic effects are compounded by the depth-dependent attenuation of near-infrared signal, which restricts reliable detection to lesions within approximately 1–2 cm of the pleural surface. As demonstrated in both clinical trials and institutional series, lesion depth is consistently the dominant determinant of fluorescence detectability, often outweighing patient-level factors or dosing parameters. Together, these findings reinforce that IMI performance is governed by fundamental physical constraints that cannot be fully overcome by probe design alone.

These considerations underscore a central principle: IMI is unlikely to replace existing imaging modalities, but rather its clinical value lies in extending detection into the intraoperative domain where conventional approaches systematically fail. High-resolution CT and FDG-PET/CT remain essential for preoperative staging and global disease assessment, while IMI extends detection into the intraoperative setting by revealing lesions that are not visible or palpable and by converting margin assessment into a real-time variable. The greatest clinical benefit is therefore realized when IMI is deployed in scenarios where conventional approaches are least reliable—namely, small, peripheral, or nonpalpable lesions, and in the assessment of margins during sublobar resection.

Taken together, IMI has the potential to shift thoracic surgery from anatomy-guided to biology-informed, in which intraoperative decision-making is increasingly driven by molecular characteristics of tissue rather than visual or tactile cues alone. However, continued integration into thoracic oncology will depend on improving TBR while remaining grounded in the biologic and optical constraints that govern fluorescence detectability. Future progress will likely be driven by multiplexed targeting, activatable probes, and computational approaches to boost TBR and interpretation. Within this framework, the role of IMI is unlikely to be universal, but rather selectively optimized for clinical scenarios in which its biologic and optical advantages can be most effectively leveraged.

## 6. Conclusions

Intraoperative molecular imaging has emerged as a promising adjunct in thoracic oncology by extending cancer detection into the intraoperative setting and enabling real-time visualization of tumor biology during surgery. Across prospective trials and large institutional experiences, IMI has demonstrated the ability to localize nonpalpable pulmonary nodules, improve intraoperative margin assessment, and identify occult synchronous malignancies that may not be detected by conventional imaging or palpation alone. These capabilities are particularly relevant in the modern era of lung cancer screening and minimally invasive surgery, where lesions are increasingly smaller, subsolid, and technically difficult to identify intraoperatively. Collectively, current evidence supports an increasingly biology-informed approach to thoracic surgery.

At the same time, the performance of IMI remains fundamentally dependent on the interaction between probe design, tumor biology, and optical physics. Lesion depth, target expression, tissue autofluorescence, and background signal continue to define the practical limits of fluorescence-guided surgery. Future progress will likely depend on the development of more selective and multiplexed imaging agents, improved imaging systems including NIR-II platforms, and computational approaches capable of enhancing real-time interpretation and quantitative analysis. Beyond technical validation, successful clinical adoption will also require practical considerations including regulatory approval, institutional investment in fluorescence-capable imaging systems, probe availability, reimbursement, and demonstration of cost-effectiveness relative to existing localization strategies. As these technologies mature, IMI has the potential to become an increasingly integrated component of precision thoracic oncology, particularly in clinical scenarios where conventional approaches remain insufficient.

## Figures and Tables

**Table 2:** Comparison of current localization strategies for pulmonary nodules in thoracic surgery.

Localization Technique	Typical Use	Advantages	Limitations	Common Complications	Role Relative to IMI	Clinical Maturity
CT-guided hook wire [89,90]	Deep/nonpalpable nodules	High accuracy	Preoperative placement	Pneumothorax, bleeding, dislodgement	Alternative	Standard
CT-guided microcoil/fiducial [89,90]	Deep nodules	Stable localization	Preoperative placement	Pneumothorax, bleeding, migration	Alternative	Standard
Radioguided surgery [91,92,93,94]	Deep nodules	Deep penetration	Radiation; logistics	Radiation logistics	Complementary	Selective
Electromagnetic navigation bronchoscopy [95,96]	Peripheral nodules	Bronchoscopic localization	Specialized equipment	Bleeding, pneumothorax	Complementary	Standard
Transthoracic dye localization [97]	Peripheral nodules	Simple, inexpensive	Dye diffusion	Pneumothorax, bleeding	Alternative	Standard
Intraoperative ultrasound [17]	Deep intraparenchymal nodules	Real-time imaging	Operator dependent	None	Complementary	Standard
Intraoperative molecular imaging	Localization, margins, occult lesions	Real-time biologic contrast	Probe- and depth-dependent	Rare adverse events; false positives	Adjunct	Emerging

Abbreviations: IMI, intraoperative molecular imaging. Note: Direct quantitative comparisons across modalities should be interpreted cautiously because reported outcomes vary according to lesion size, depth, patient selection, institutional expertise, and study methodology.

## Data Availability

No new data were created or analyzed in this study. Data sharing is not applicable to this article.

## Abbreviations

The following abbreviations are used in this manuscript:

IMI	Intraoperative molecular imaging
ICG	Indocyanine green
NLST	National Lung Screening Trial
TBR	Tumor-to-background ratio
SBR	Signal-to-background ratio
